# A Synbiotic with Tumor Necrosis Factor-*α* Inhibitory Activity Ameliorates Experimental Jejunoileal Mucosal Injury

**DOI:** 10.1155/2018/9184093

**Published:** 2018-05-10

**Authors:** Ryoki Takahashi, Takayasu Noguchi, Yoko Mizoguchi, Tadashi Shimoyama, Teruko Nakazawa, Tohru Ikuta

**Affiliations:** ^1^General Medicine and Community Health Science of the Sasayama Medical Center, Hyogo College of Medicine, Sasayama, Hyogo 669-2300, Japan; ^2^Sagami Research Laboratory, Kanagawa 252-0231, Japan; ^3^Department of Gastroenterology, Hirosaki University Graduate School of Medicine, Hirosaki, Aomori 030-8563, Japan; ^4^Department of Microbiology, Yamaguchi University School of Medicine, Ube, Yamaguchi 755-8505, Japan; ^5^Department of Anesthesiology & Perioperative Medicine, Medical College of Georgia, Augusta University, Augusta, GA 30912, USA

## Abstract

Despite the recent development of biological modifiers for inflammatory bowel diseases (IBD), there continues to be considerable interest in fermented medicines because of its negligible adverse effects. We previously showed that the synbiotic Gut Working Tablet (GWT) alleviates experimental colitis. Here we show that GWT is capable of ameliorating jejunoileal mucosal injury, which is frequently seen with IBD. We created experimental jejunoileal mucositis in rats by injection of methotrexate (MTX) which increases intestinal permeability, a hallmark finding of IBD. Administering GWT to MTX-injected rats restored intestinal integrity by reversing villi shortening, crypt loss, and goblet cell depletion in the mucosa. Also GWT reduced activities of myeloperoxidase and lipid peroxidase and increased superoxide dismutase activity, which is critical for maintaining intestinal function. We further found that GWT suppressed mRNA expression of tumor necrosis factor-*α* (TNF-*α*) and interleukin-12 (IL-12) in macrophage and reduced TNF-*α* mRNA expression in specimens with experimental colitis, which is in contrast to VSL#3 that enhanced TNF-*α* production. Together, the current and previous animal studies clearly demonstrate the protective role of GWT in chemically induced enterocolitis. Crohn's disease, a well-known IBD, can affect any portion of the intestine, and these results suggest that GWT may be useful as a novel therapeutic or maintenance therapy for IBD.

## 1. Introduction

Inflammatory bowel disease (IBD), encompassing Crohn's disease (CD) and ulcerative colitis, is characterized by acute and chronic inflammatory mucosal injury of the gastrointestinal tract that is associated with various levels of ulceration [1]. Although significant progress has been made in determining the pathogenesis of this disorder [2], the clinical manifestations and pathological findings are extremely heterogeneous and complex, making it difficult to clarify the molecular and physiologic mechanisms underlying this disorder. Several experimental models for IBD have been developed in various animal species [3], and multiple genetic abnormalities including immune and inflammatory systems [4] and environmental factors, most notably intestinal microflora [5], are likely involved in the initiation, progression, and complications of IBD. Previous studies investigating the immunologic aberrations underlying the disorder have shown that the helper T-cell-mediated cellular and humoral immunities may be deviant [2]. More importantly, in patients with IBD, tumor necrosis factor-*α* (TNF-*α*) is overexpressed in the monocytes and macrophages that infiltrate the mucosa; there are, however, substantial differences between CD and ulcerative colitis in the level and localization of monocyte infiltration [4, 6]. TNF-*α* is elevated in the intestinal mucosa of patients with IBD and clinical trials have found that anti-TNF-*α* antibodies significantly improve clinical manifestations of IBD [7–9]. Relevant to these findings, mutations of interleukin-10 (IL-10) receptors whose deficient IL-10 signaling resulted in elevated TNF-*α* production were discovered in pediatric IBD studies [10, 11].

Despite recent developments in novel therapeutics including anti-TNF-*α* antibodies for IBD [12], interest continues to be directed toward probiotics, prebiotics, or fermented medicines as either a treatment modality or maintenance therapy. This is likely because anti-TNF-*α* antibodies have various adverse effects [13] and increase the potential for malignancy [14], while synbiotics, which are a combination of probiotics and prebiotics, have negligible adverse effects [15]. Moreover, inflammation of the gastrointestinal tract is substantially regulated by intestinal microflora [16, 17] and therapies utilizing anti-inflammatory agents, immunosuppressants, and monoclonal antibodies are not capable of modulating intestinal microflora [5]. A number of clinical trials on the use of synbiotics for ulcerative colitis and CD have been conducted [5]. Gionchetti et al. demonstrated the substantial clinical efficacy of VSL#3, which is comprised of probiotics, as maintenance therapy for chronic relapsing pouchitis [18, 19]. Shen et al., however, were unable to confirm the clinical effectiveness of VSL#3 in antibiotic-dependent patients with pouchitis [20]. Although the efficacy of synbiotics in ulcerative colitis has been well studied, its efficacy in CD remains less clear because a relatively small number of patients were enrolled in the trials [5, 21].

While developing novel and effective combinations of synbiotics to treat CD, we previously showed that Gut Working Tablet (GWT), which includes both probiotics and prebiotics and is hence a synbiotic, alleviated experimental colitis induced by trinitrobenzene sulfonic acid (TNBS) in rats [22]. In contrast to VSL#3, GWT is composed of the fermentation products of several cereal germs with the* Aspergillus oryzae* strain NK (*A. oryzae* NK-Koji), a lactic acid bacterium,* Enterococcus faecium* and its fermentation products, and* Saccharomyces cerevisiae*, otherwise known as dried brewer's yeast [22]. Recently, we also found that GWT has beneficial effects on constipation by restoring the concentration of short chain fatty acids in the intestine [23]. In this study, we first examined physiological effects of GWT on experimental jejunoileitis. As jejunoileitis is frequently seen in CD patients [1, 24], we induced experimental jejunoileal mucosal injury in rats by methotrexate (MTX). MTX induces intestinal mucosal injury by increasing intestinal permeability [25, 26]; elevated intestinal permeability is a major pathophysiological finding in IBD [27–29]. After administering GWT, the pathophysiological parameters of jejunoileal mucosal injury were improved significantly. Furthermore, we found that GWT significantly suppresses the production of cytokines such as TNF-*α* and interleukin-12 (IL-12) in macrophages and in the colon of rats with TNBS-induced colitis. These studies demonstrate the ability of GWT to alleviate chemically induced experimental enterocolitis, presumably in part by suppressing the expression of TNF-*α* and IL-12. Because anti-TNF-*α* antibodies are associated with various adverse effects [14, 30], GWT may represent a potentially safer therapeutic agent for the various types of enterocolitis associated with CD or ulcerative colitis, both of which are well-known IBD.

## 2. Materials and Methods

### 2.1. Composition of GWT

GWT was kindly provided by Wakamoto Pharmaceutical Co. Ltd. (Tokyo, Japan) as described previously [22, 23].

### 2.2. Animal Study of Jejunoileal Mucosal Injury

**Figure 1:**
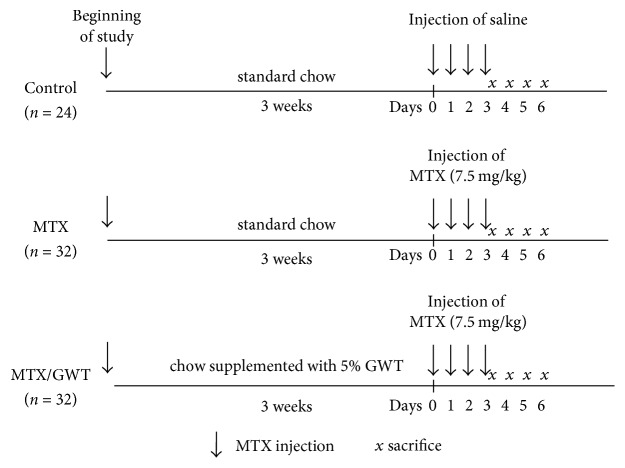
Experimental scheme of MTX-induced jejunoileal mucosal injury in rats. Controls (*n* = 24) were given standard chow (CE-2) for 3 weeks and sacrificed 3 to 6 days later. MTX (*n* = 32) and MTX/GWT (*n* = 32) groups were given CE-2 or CE-2 supplemented with 5% GWT for 3 weeks, injected with MTX (7.5 mg/kg) for 4 consecutive days from day 0 to day 3 (shown by downward arrows), and sacrificed on the days marked with an *x*.

Male Sprague-Dawley (SD) rats (Charles River Laboratories, Yokohama, Japan) were housed individually as described previously [22]. The animal study was performed in accord with the guidelines of the Japanese Association for Laboratory Animal Science and the animal protocol (protocol # IBD-40) was reviewed and approved by the Animal Ethics Committee of Sagami Research Laboratories (Kanagawa, Japan). All rats were fed CE-2 (Nihon CLEA, Tokyo, Japan), a standard commercial chow powder, and water ad libitum during a 3-day acclimatization period. As shown in Figure 1, 5-week-old rats were separated into 3 groups: control (*n* = 24), MTX (*n* = 32), and MTX/GWT (*n* = 32). For 3 weeks, MTX rats were fed CE-2 and MTX/GWT rats were fed CE-2 containing 5% GWT; the GWT had no effect on daily food intake or body weight control. Rats in the MTX and MTX/GWT groups were then injected with MTX (7.5 mg/kg, Calbiochem, La Jolla, CA, USA) intraperitoneally on days 0, 1, 2, and 3. Rats in the control group were fed CE-2 and injected with a volume of saline (0.9% NaCl).

### 2.3. Preparation of Jejunoileal Specimens for Studying Enzyme Activities and Histological Procedures

Rats were sacrificed on days 3, 4, 5, and 6 by cervical dislocation after being anesthetized with nitrogen gas (Figure 1). A distal segment (jejunoileum) of the small intestine was removed and a proximal 5 cm segment (3 to 8 cm from pylorus) was used to measure thiobarbituric acid-reactive substances (TBARS), myeloperoxidase (MPO), and superoxide dismutase (SOD) activity as described [22]. Lipid peroxidation was determined by measuring malondialdehyde as TBARS using a commercial kit (Wako Pure Chemical Ind. Ltd., Wako, Japan) and expressed as nmol/g tissue. Intestine specimens were fixed in 10% buffered formaldehyde and then immersed in 20% sucrose-PBS and stained with hematoxylin and eosin (HE) or periodic acid Schiff (PAS). Heights of six well-oriented villi (three villi each in two sections) and the numbers of crypts in two randomly selected areas (900 *μ*m × 700 *μ*m) on HE-stained samples were measured by using an image analysis apparatus (Adobe Photoshop). In addition, histologic damage scores were determined by using HA-stained jejunoileal specimens according to the methods of Shimizu et al. [31]. Briefly, scores of histologic damage were defined as the sum of the individual scores graded as 0 (none), 1 (mild/moderate), or 2 (severe) for each of the following three microscopic findings: inflammatory cell infiltration, edema or hyperplasia, and mucosal disappearance in specimens. Goblet cells in two randomly selected crypts were counted on PAS-stained samples.

### 2.4. Measurement of Mucosal Protein and DNA

A distal segment (jejunoileum) of the small intestine was opened and washed in ice-cold saline with gentle agitation. After the mucous layer was scraped with a slide glass and weighed, the protein and DNA contents were determined by BCA Protein Assay Kit (Pierce, ThermoFisher Scientific Inc., Rockford, IL, USA) and Schneider's method [32], respectively. Mucosal weight was expressed as milligrams per centimeter (mg/cm) tissue and protein and DNA contents as milligrams per gram (mg/g) of jejunoileal mucosa.

### 2.5. Effects of GWT Extracts on Cytokine Synthesis in Macrophages and in Experimental Colitis

To prepare GWT extracts, 10 g of GWT powder was dissolved in 10 mL of phosphate-buffered saline by shaking for 30 min at room temperature. The supernatant was isolated and sterilized by filtering through a 0.45 *μ*m membrane (Millipore, Billerica, MA, USA). The protein concentration of the extracts was adjusted to 4 mg/mL. Murine macrophage cells (RAW264.7, American Type Culture Collection, Manassas, VA, USA) were cultured as described previously [22]. Five thousand RAW264.7 cells were placed in 96-well microplates and cultured overnight. Various concentrations of lipopolysaccharide (LPS) or GWT extracts were added to the wells. Supernatants were harvested 24 hrs after the addition of LPS or GWT and cytokine levels were determined by ELISA assays (Biosource, Grand Island, NY, USA) according to the manufacturer's protocols. To determine the effect of GWT on TNF-*α* synthesis in experimental colitis, male 3-week-old rats were divided into three groups: control (*n* = 3), TNBS (*n* = 3), and TNBS/GWT (*n* = 3). For 4 weeks, the control and TNBS groups were fed AIN-93M (Nihon CLEA, Tokyo, Japan), while those in the GWT and TNBS/GWT groups were fed AIN-93M containing 5% GWT. To induce colitis, 250 *μ*L of 30 mg TNBS (Fluka, St. Louis, MO, USA) dissolved in 50% of ethanol was instilled into the colon. The rat colons were harvested 7 days later. Mononuclear cells were isolated by mincing the specimens and extracting total RNA from cells. TNF-*α* expression levels were determined by real-time PCR as described [22] using the following primers: TNF-*α* (NM012675.3), 5′-CGAGTGACAAGCCCGTAGCC- 3′ and 5′-GGATGAACACGCCAGTCGCC-3′; Glyceraldehyde-3-phosphate dehydrogenase (GAPDH), 5′-TCCCTCAAGATTGTCAGCAA-3′ and 5′-AGATCCACAACGGATACATT-3′. The relative levels of TNF-*α* PCR products were presented as a ratio of the mRNA levels to that of GAPDH from the same sample.

### 2.6. Statistical Analysis

The Mann–Whitney *U* test was applied to determine significance levels between two of the three groups using SPSS software version 15.0 (Statistical Package for Social Sciences for Windows, SPSS Inc., Chicago, IL, USA). Data were expressed as means ± SEM, and values of *p* < 0.05 were considered to be statistically significant.

## 3. Results

### 3.1. Effects of GWT on Bodyweight and Food Intake in Rats Injected with MTX

**Figure 2:**
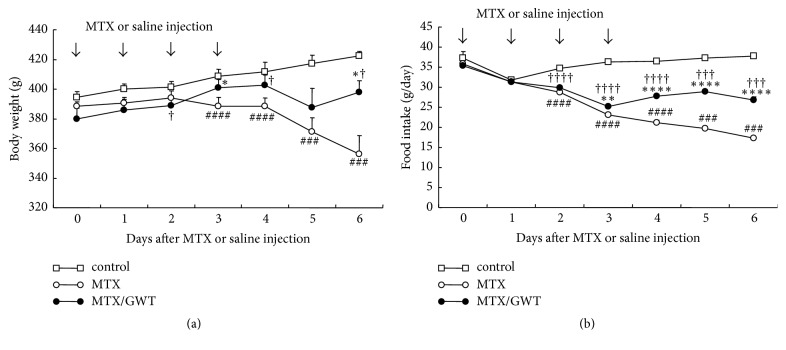
Effects of GWT on body weight (a) and food intake (b) of MTX-injected rats. The rats in the MTX group (open circles) were given standard chow (CE-2) and injected with MTX (7.5 mg/kg, i.p). The rats in the MTX/GWT group (closed circles) were given 5% GWT-supplemented chow and injected with MTX, while the rats in the control group (open squares) were fed standard chow and injected with saline. Arrows indicate MTX or saline injection. Values are the mean ± SE values. In this and subsequent figures, the statistical symbols are as follows: ^*∗*^*p* < 0.05; ^*∗∗*^*p* < 0.01; ^*∗∗∗*^*p* < 0.005; ^*∗∗∗∗*^*p* < 0.001 (between the MTX and the MTX/GWT group); ^#^*p* < 0.05; ^##^*p* < 0.01; ^###^*p* < 0.005; ^####^*p* < 0.001 (between the MTX and the control group); and ^†^*p* < 0.05; ^††^*p* < 0.01; ^†††^*p* < 0.005; ^††††^*p* < 0.001 (between the control and the MTX/GWT groups).

We previously showed that CE-2 supplemented with 5% GWT exerts significant protective effects against TNBS-induced colitis [22]. To further dissect the physiological effects of CE-2 with 5% GWT on enterocolitis, we induced experimental jejunoileitis by injecting MTX (7.5 mg/kg bodyweight) intraperitoneally into rats in the MTX and MTX/GWT groups for 4 consecutive days following a 3-week acclimatization period (Figure 1). MTX is shown to elicit intestinal mucositis by increasing intestinal permeability [25, 26]. It appears that MTX induces pathophysiological conditions that mimic those seen with IBD as elevated intestinal permeability is a hallmark of this disorder [27–29]. During the acclimatization period, the average bodyweight (Figure 2(a)) and food intake (Figure 2(b)) were comparable among the three groups of rats, irrespective of whether they were consuming standard chow (CE-2) or CE-2 with 5% GWT, suggesting that GWT supplementation does not affect rat bodyweight. After MTX injection, however, the bodyweight of rats in the MTX group began to decrease on day 3 and thereafter (Figure 2(a)). GWT supplementation significantly reduced the loss of bodyweight on days 3 (*p* = 0.020) and 6 (*p* = 0.027) (MTX/GWT versus MTX group; Figure 2(a)). However, the bodyweights of rats in the MTX/GWT group were still lower than those of the control group (*p* = 0.039, 0.014, and 0.020 on days 2, 4, and 6, resp.; Figure 2(a)). Similarly, animals in both the MTX and MTX/GWT groups showed a significant reduction in food intake as compared with the control group on days 2 to 6 (*p* < 0.005), but the MTX/GWT group had significantly improved food intake compared to the MTX group on days 3 to 6 (*p* < 0.01) (Figure 2(b)). These results suggest that GWT ameliorates the physiological effects on bodyweight and food intake induced by MTX injection.

### 3.2. Effects of GWT on Intestinal Mucosa of Rats Injected by MTX

**Figure 3:**
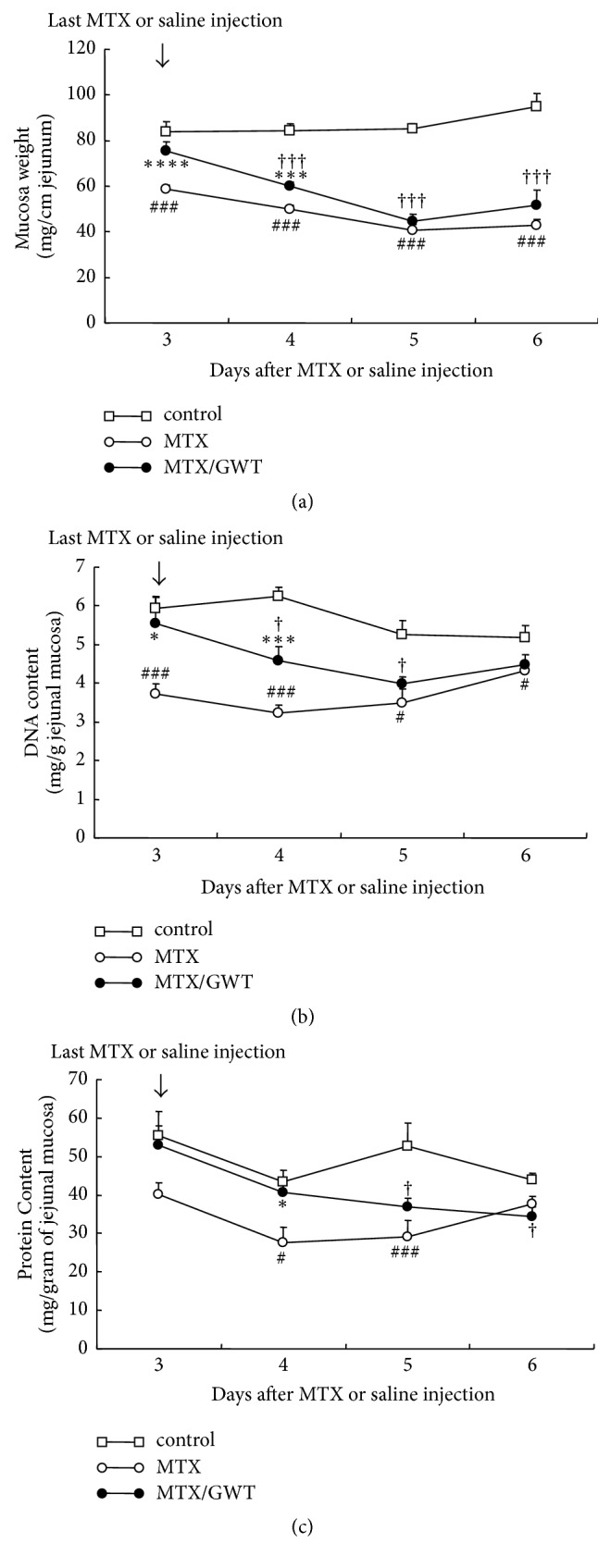
Effects of GWT on intestinal mucosa of rats injected by MTX. Mucosal weight (a), DNA (b), and protein (c) contents in the ileum of MTX-treated rats were examined. The numbers of rats in the MTX, MTX/GWT, and control are described in Figure 1 legend. Values are the mean ± SE. Statistical symbols are shown in Figure 2 legend.

A major lesion in the gastrointestinal tract induced by MTX includes the jejunoileum [33–35]. To determine whether GWT can protect the integrity of the jejunoileum from MTX-induced intestinal damage, we measured mucosal weight and protein and DNA contents of rat tissue in the three experimental groups (Figure 3). As compared with the control group, the mucosal weight (Figure 3(a)), DNA (Figure 3(b)), and protein (Figure 3(c)) contents of the MTX group were significantly decreased on day 3 and thereafter: mucosa, *p* = 0.002; DNA content, *p* = 0.002; protein content, *p* = 0.03. On day 4, rats in the MTX/GWT group had significantly higher mucosal weight (*p* = 0.001), DNA content (*p* = 0.004), and protein content (*p* = 0.04) compared to the MTX group. However, such improved physiological parameters were not observed on days 5 and 6. These results demonstrate that MTX is detrimental to protein and DNA synthesis in the ileum, which is consistent with other studies [34, 35]. Further, the administration of GWT-supplemented CE-2 to rats injected with MTX significantly protected animals from loss of body weight, which is presumably a consequence of improved food intake, but its effects on the loss of mucosal weight and DNA and protein contents of the jejunoileum are limited to a certain stage of MTX-induced mucosal damage.

### 3.3. Histologic Analysis with Effects of GWT on MTX-Induced Jejunoileal Injury

**Figure 4:**
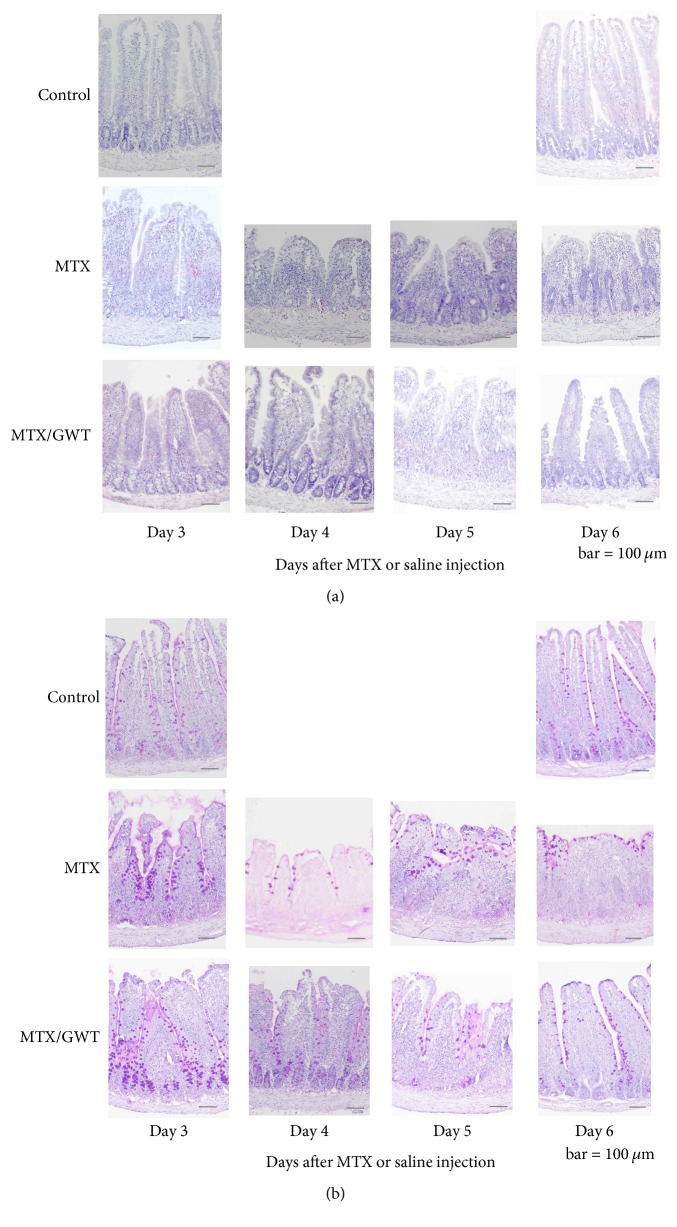
Histologic analysis with effects of GWT on MTX-induced jejunoileal injury. Jejunoileal specimens of rats from the 3 groups were stained by HE (a) and PAS (b) (magnification, ×100). The rats in the MTX group were fed standard chow (CE-2) and injected with MTX four times. The rats in the MTX/GWT group were fed 5% GWT-supplemented chow and similarly injected with MTX, while the rats in the control group were fed standard chow and injected with saline. Rats were sacrificed on days 3, 4, 5, and 6 after the first MTX injection. (a) Compared to the control group (top row), MTX treatment (middle row) led to extensive structural damage including villus shortening, atrophy, desquamation of surface epithelium, cystic dilatation in crypt, and crypt loss. Such changes were improved in the MTX/GWT group (bottom row). (b) PAS staining reveals that a majority of goblet cells in the MTX-treated group specimens (middle row) were lost from the crypts, while a significant number of goblet cells were preserved in the MTX/GWT group specimens (bottom row).

**Figure 5:**
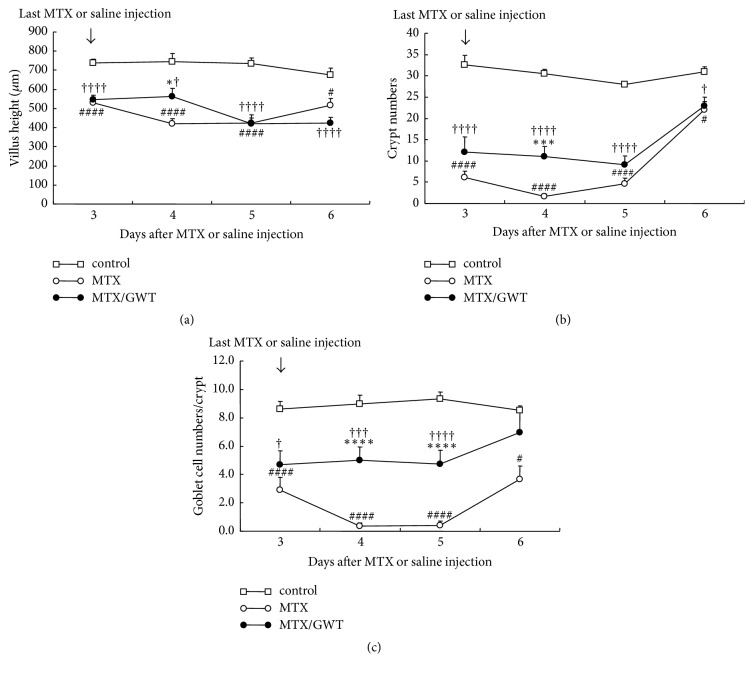
Quantitative analysis with villus height (a), crypt numbers (b), and goblet cell numbers (c) in the jejunoileum of rats from the control, MTX, and MTX/GWT groups. The number of rats per group is shown in Figure 1 legend. Values are the mean ± SE. Statistical symbols are shown in Figure 2 legend.

Next, we performed histologic analyses to define the effect of GWT on MTX-induced jejunoileal mucosal injury. A middle segment of the small intestine was taken from animals from each of the three groups and stained by HE and PAS. Compared to the control group specimens, MTX treatment resulted in extensive structural damage including villus shortening, atrophy, desquamation of surface epithelium, cystic dilatation in crypt, and crypt loss (Figure 4(a) top and middle rows). These histologic changes were significantly improved in the MTX/GWT group specimens (Figure 4(a) bottom row). The number of goblet cells was then examined by PAS staining (Figure 4(b)). On day 4 and thereafter, the MTX group specimens showed an almost total loss of goblet cells in the crypts and only a few were seen in the apex (Figure 4(b) middle row), while a significant number of goblet cells were preserved in the MTX/GWT group specimens (Figure 4(b) bottom row). To verify these pathological observations, villus height and number of crypt and goblet cells were analyzed (Figure 5). MTX treatment caused a significant reduction in the villus height on day 3 and thereafter (Figure 5(a)). GWT supplementation (MTX/GWT group) increased villus height only on day 4 (*p* = 0.023 versus MTX), but there were no significant differences for other days, suggesting that GWT has limited protective effects on the intestinal villous structure. MTX treatment resulted in extensive crypt loss on days 3 to 5; however, the crypt loss on day 4 was significantly diminished in the rats given GWT (*p* = 0.001 versus MTX; Figure 5(b)). Further, on days 4 and 5, goblet cells were almost entirely absent in the MTX group specimens, but almost half of the goblet cells were retained in the MTX/GWT group specimens (*p* < 0.001 versus MTX; Figure 5(c)). The number of goblet cells was similar in the MTX/GWT and control groups, indicating that goblet cells are efficiently preserved by the administration of GWT in MTX-injected rats. To accurately determine whether GWT protected the jejunoileum from MTX-induced mucosal injury, we calculated histologic damage scores in the specimens of the control, MTX, and MTX/GWT groups (Figure 4(a)). The results are shown in Table 1. No damage was detected in any of the rats in the control group through up to day 6. Next, we compared the histologic damage scores between the MTX and MTX/GWT groups. The scores of the MTX/GWT group were significantly improved on days 4 and 5 compared to those of the MTX group. This observation appears to be compatible with the results of mucosa weight, DNA content, protein content (Figures 3(a)–3(c)), and villus height (Figure 5(a)). The improved food intake of the MTX/GWT group may have positive consequences on the recovery of MTX-injured mucosa.

### 3.4. Effects of GWT on MPO, Lipid Peroxidation, and SOD Activities

**Figure 6:**
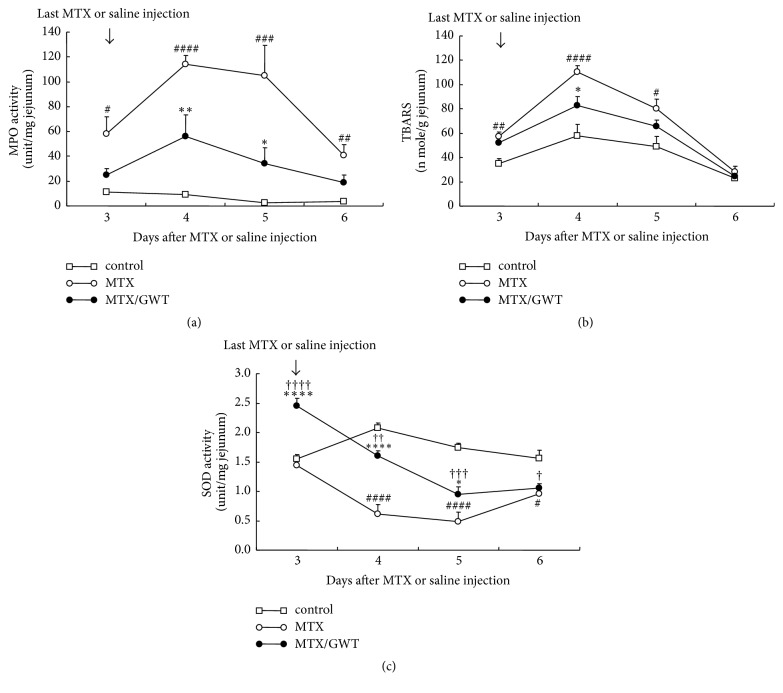
Effects of GWT on MPO, lipid peroxidation, and SOD activity. Changes in MPO activity (a), TBARS levels (b), and SOD activity (c) in the ileum of rats from the control, MTX, and MTX/GWT groups. The number of rats per group is shown in Figure 1 legend.

MTX injection alters the activity of several enzymes in intestinal mucosal tissues, some of which are relevant to oxidative stress [35–37]. As shown in Figures 6(a) and 6(b), both jejunoileal lipid peroxidation and MPO activity in MTX-treated rats were significantly increased when compared to the control rats (*p* < 0.05 to 0.001). An increase in MPO activity is likely associated with neutrophil infiltration and preceded by lipid peroxidation due to the production of reactive oxygen species. However, GWT supplementation decreased MPO activity on days 4 and 5 (Figure 6(a), *p* = 0.01 on day 4, *p* = 0.02 on day 5) and on TBARS on day 4 (Figure 6(b), *p* = 0.029). In contrast, SOD activity was markedly decreased on days 4 to 6 in the MTX-treated rats compared with controls (Figure 6(c), *p* < 0.05 to 0.001). It is striking that the SOD activity on day 3 in the MTX/GWT group was significantly higher than that of the controls (*p* = 0.001), presumably due to the induction of SOD enzymes by some unidentified components in GWT. In a separate study, when wild-type rats were given CE-2 fortified with 5% GWT, jejunoileal SOD activity was significantly elevated compared to that of wild-type rats given CE-2 only (*p* < 0.05 to 0.001, data not shown). Accordingly, the SOD activity in the MTX-treated group was markedly decreased on days 4 and 5, but that of the MTX/GWT-treated group was significantly higher than those of the MTX group (*p* < 0.001 on days 3, 4 and *p* = 0.046 on day 5; Figure 6(c)). Together, these results demonstrate that GWT not only is able to reduce MOP activity and lipid peroxidation, but also is able to increase SOD activity in the ileum of rats, perhaps contributing to the reduction in oxidative stress that had been enhanced by MTX.

### 3.5. GWT Reduces Cytokine Production in Macrophages and in Experimental Enterocolitis

**Figure 7:**
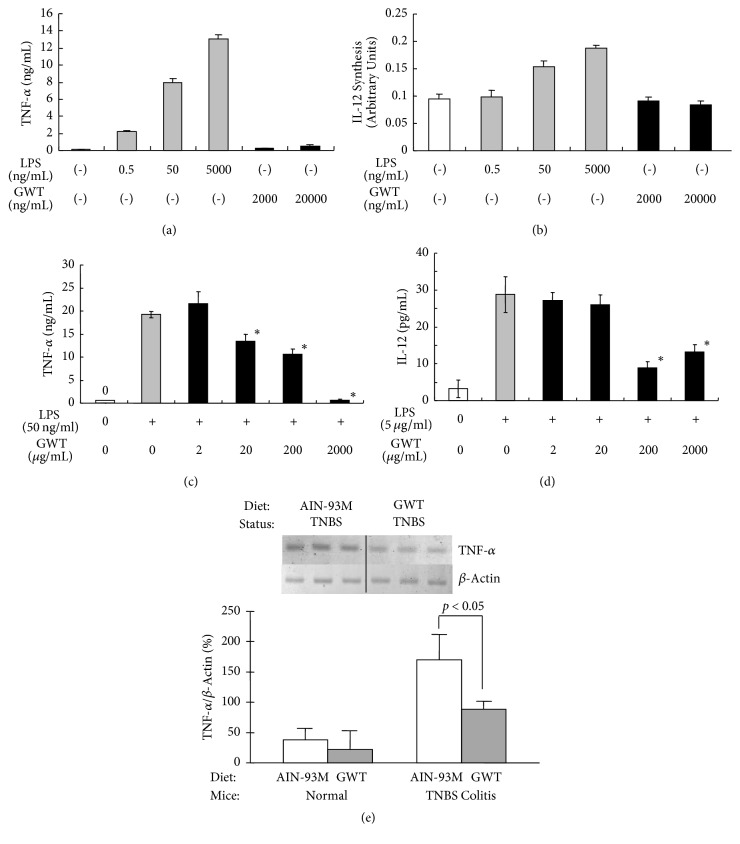
GWT reduces cytokine production in macrophages and experimental enterocolitis. Effects of GWT on the production of TNF-*α* (a and c) and IL-12 (b and d) in murine macrophages (RAW264.7) and those stimulated with LPS were examined. Cytokine levels were determined by ELISA assays. (e) Effects of GWT on TNF-*α* expression were examined in intestinal mucosa with TNBS-induced colitis. Rats were given either AIN-93M or AIN-93M including 5% GWT, shown as GWT, for 2 weeks. TNF-*α* expression levels in mononuclear cells in intestinal mucosa were determined by RT-PCR. The means of TNF-*α* mRNA levels of each group are shown by horizontal bars.

To further investigate the molecules involved in the GWT-mediated improvement of experimental intestinal mucosal injury, we examined whether prepared GWT extracts would increase the production of TNF-*α* and IL-12, two cytokines believed to be involved in the pathogenesis of CD, in macrophage cells [6, 38, 39]. We found that whereas LPS induced the synthesis of both cytokines (Figures 7(a) and 7(b)), GWT extracts instead suppressed the synthesis of TNF-*α* (Figure 7(c)) and IL-12 (Figure 7(d)) in macrophages stimulated by LPS. Next we examined the effect of GWT on TNF-*α* expression in mononuclear cells that are infiltrated in colonic tissues with experimental colitis. The expression TNF-*α* mRNA in mononuclear cells derived from TNBS-induced colitis was about 4 times higher than that of cells prepared from normal colonic tissues (Figure 7(e)), and this is consistent with our current understanding of the molecular mechanisms that play a role in CD [4]. However, administration of GWT to rats with TNBS-induced colitis reduced TNF-*α* mRNA expression by about 50%, indicating that GWT significantly inhibits TNF-*α* mRNA expression in vivo.

## 4. Discussion

Several lines of preclinical and clinical evidence indicate that intestinal microflora play a role in the pathogenesis and severity of IBD [16, 17, 40, 41]. Here we focused on investigating the roles of synbiotic mixtures in IBD as they would have fewer adverse effects than other currently available therapeutics [2, 9, 42]. We previously showed that the synbiotic GWT alleviates TNBS-induced colitis [22]. CD can induce inflammation in various portions of the gastrointestinal tract, including the ileum and the colon. In this study, MTX injections triggered jejunoileitis in SD rats which in turn elicited marked histological alterations and an increase in MPO activity. These effects are consistent with those reported by Carneiro-Filho et al. [26]. As stated earlier, intestinal permeability is increased by MTX injection [25]. Hence, MTX injection likely creates pathophysiological conditions in the intestine that are similar to those observed with IBD [29]. We found here that, compared to animals injected with MTX, those that received both MTX and GWT had (1) higher protein and DNA content (except for day 6; Figure 3), (2) higher villous height and mucosal weight as well as more preserved crypts and goblet cells (Figures 4 and 5), and (3) reduced levels of MPO activity and oxygen stress, as measured by TBARS and SOD assays (Figure 6). That there were no significant differences in some histological or enzymatic parameters between the MTX and MTX/GWT groups on day 6 may be attributable to the initiation of regenerative processes for damaged tissues [25]. Furthermore, while VSL#3 increased the production of the proinflammatory cytokine TNF-*α* in SAMP mice [43], we found that GWT inhibited the production of TNF-*α* and IL-12 in macrophages stimulated with LPS as well as TNBS-induced colitis (Figure 7). Taken as a whole, this study demonstrates that GWT is able to sustain some integrity of the jejunoileal mucosa against acute mucositis induced by MTX, as shown in our previous study [22], that GWT is useful for treating jejunoileitis and colitis, two typical lesions of CD.

While we found substantial protective effects of GWT on chemically induced colitis [22] and jejunoileitis in our animal studies, the probiotic agent VSL#3 showed no significant effects on a similar experimental colitis induced by dinitrobenzene sulfonic acid [44]. VSL#3, which consists of lyophilized bacteria such as* Lactobacillus*,* Bifidobacterium*, and* Streptococcus salivarius* [19], also generated a mixed response in IBD clinical trials [18, 45]. In contrast, Lactobacilli-fermented oatbase, a component of GWT, exhibited strong ameliorating effects on the severity of MTX-induced colitis [46]. A major component of GWT is the fermentation products of several cereal germs including* A. oryzae* NK-Koji and* E. faecium*; therefore, it may be that the fermented products of probiotics have physiologically robust effects on ileitis and colitis associated with IBD. In order to identify the components that are critical for the clinical efficacy of GWT, we gave individual components of GWT to patients with CD. Although* A. oryzae* NK-Koji was effective in alleviating clinical manifestations, neither* E. faecium* nor* S. cerevisiae* exerted any clinical benefits (R. Takahashi, unpublished observation). These clinical observations further suggest the notion that fermentation products can substantially ameliorate CD symptoms.

The molecular mechanisms by which VSL#3 and GWT ameliorate the clinical severity of IBD are not fully understood [47]; this is largely because the etiology of CD is not fully understood [1, 17]. Presumably, synbiotics modulate various aspects of the pathophysiology of CD through multiple physiological or molecular mechanisms. First, in IL-10-deficient mice, an animal model of IBD, the probiotic bacteria compound VSL#3 enhances the barrier function of intestinal epithelial cells [3], which may be mediated in part by inhibiting the secretion of proinflammatory cytokines such as TNF-*α* and interferon-*γ* [48]. More importantly, as shown previously [22], GWT extracts inhibited LPS-induced production of TNF-*α* in monocyte-like cells and downregulated the expression of interleukin-1*β* (IL-*β*) in colon tissue. Here we further confirmed that GWT suppresses TNF-*α* production in intestinal mucosa with TNBS-induced colitis (Figure 7(e)). TNF-*α*, a critical cytokine that mediates inflammation associated with CD [4, 6], is regulated by IL-1*β* [49]. It is possible that synbiotics such as GWT can restrain the inflammation elicited by TNF-*α* and IL-1*β* in CD. The inhibitory effects of GWT on these cytokines, which are potent chemoattractants for neutrophils [50], may be involved in the reduced MPO activity observed in the MTX/GWT group rats (Figure 6(a)). In contrast, in an animal model, VSL#3 downregulated chemokine signaling pathways [51] and upregulated the intestinal barrier systems at the mucosal surface [52, 53] but enhanced TNF-*α* production in primary cell cultures [43]. This suggests that the molecular mechanisms by which GWT alleviates the pathophysiology of CD could be distinct from those by VSL#3. Second, previous studies utilizing chemically induced enterocolitis [3] showed that increased oxidative stress likely plays a major role in developing mucosal injury of the gastrointestinal tract in IBD [37, 42, 54]. SODs likely constitute an important antioxidant defense system and their activity is reduced in TNBS- and MTX-induced enterocolitis, as shown in this (Figure 6(c)) and previous studies [22]. Importantly, the SOD activity of rats in the MTX/GWT group was significantly higher than those in the MTX group, with the exception of day 6 (Figure 6(c)), suggesting that GWT is capable of enhancing SOD activities in intestinal mucosa. This view is supported by our recent finding that rats fed GWT-supplemented chow had significantly higher SOD activity (2.5 to 3.0 units/mg tissue) than those fed standard chow (1.5 units/mg tissue), regardless of MTX treatment (R. Takahashi, unpublished observation). Together, GWT may alleviate clinical manifestations of CD by (1) decreasing the expression of inflammatory cytokines such as TNF-*α* and IL-1*β* as well as of IL-12, which is increased in this disorder and enhances T helper 1 response [38], and (2) strengthening the antioxidant defense system by increasing SOD activity, which is presumably necessary to prevent MTX-induced oxidative damage.

Collectively, our animal experiments demonstrate that GWT exerts some protective effects on MTX-induced jejunoileal injury in rats. Also, the results from this study and our previous animal study [22] are encouraging and warrant further investigation into the clinical efficacy of GWT in CD. The advent of anti-TNF-*α* antibodies has revolutionized the treatment of IBD, but some reports suggest an increased risk of malignancy and other bacterial or viral infections [9, 14, 30]. Further, patients with IBD exhibit variable responses to such antibodies [55]. Our studies suggest that GWT may be a useful therapeutic agent in treating IBD, especially in combination with other chemicals such as immunosuppressants and anti-TNF-*α* antibodies, because GWT is capable of suppressing the production of IL-1*β* and TNF-*α* in intestinal tissues [22]. GWT may also be particularly useful for the treatment of CD because GWT likely alleviates the inflammation associated with both jejunoileitis and colitis, two major symptoms of the disorder.

## 5. Conclusions

Our studies demonstrate that the synbiotic GWT improves the pathophysiology of chemically induced enterocolitis, which may be attributable in part to the inhibition of TNF-*α* and IL-12 production. In contrast to VSL#3, it is possible that GWT is able to alleviate the clinical severity of CD through mechanisms that are relevant to those of biological agents such as anti-TNF-*α* antibodies.

## Figures and Tables

**Table 1:** Histologic damage scores of jejunoileal specimen (mean ± SD).

Day after MTX	Group		
	MTX	MTX/GWT	*p* value
Day 3	4.0 ± 1.0	3.1 ± 1.2	0.139
Day 4	5.4 ± 0.7	3.4 ± 1.7	0.015^*∗*^
Day 5	4.5 ± 0.9	3.6 ± 0.7	0.046^*∗*^
Day 6	4.1 ± 1.5	4.1 ± 1.5	1.000

^*∗*^ *p* < 0.05 between MTX and MTX/GWT.
